# *In silco* analysis of carboxamide derivatives of piperidine as potential antagonists of CCR5

**DOI:** 10.1186/1471-2334-14-S3-E21

**Published:** 2014-05-27

**Authors:** A Harishchander, J Michael Joshua, U Benjamin Jason, D Alex Anand

**Affiliations:** 1 Department of Bioinformatics, Sathyabama University, Chennai, India

## Background

Analysis and identification of competitive antagonist in the co receptor binding of HIV is a challenging task towards overcoming the tropism of HIV. Hence analyzing the structure activity relationship between CCR5 and the derivatives of carboxamide is an initiation towards discovering the future inhibitors of CCR5.

## Methods

We have identified the interaction between CCR5 and existing sulphonyl derivatives of carboxyl amine and it was followed by construction of various derivatives of carboxamide with trans confirmation of carboxyl phenol. Further, the constructed molecules were screened on the basis of various molecular descriptors. The potential derivates were identified using molecular dynamics simulations in 1 nano second using Discovery Studio software suite (version 2.0).

## Results

Regression Analysis of various molecular descriptors showed a threefold increase in the competitive binding of carboxamide derivatives with CCR5 and the increased activity of existing sulphonyl derivatives is single fold. Binding Energies of carboxamide derivatives were less than 2 KCal of sulphonyl derivatives. Conformational analysis using Molecular Dynamics Simulation of sulfo carboxamide derivatives confirms a twofold increased stability when compared with sulphonyl derivatives of piperidine.

## Conclusion

Construction of analogs based on sulfo carboxamide using chemsketch generated fourteen compounds. *In silico* analysis of these piperidine based Sulfo carboxamide derivates show a better competitive inhibition than existing sulphonyl derivatives of carboxyl amine. Further *in vivo* tests are required to analyze the effective concentration for constructing effective derivatives of carboxamide based piperidine as potential antagonists of CCR5 in future.

